# Platina and Its Accompanying Metals

**Published:** 1860-04

**Authors:** 


*Platina and its Accompanying Metals.*—We give in this number the commencement of an elaborate and able memoir on this subject by the well known French Metallurgists, St. Claire Deville and Debray. Although much of the detail given may appear to be of merely scientific interest, yet we have preferred to furnish our readers with the memoir in full, as information is needed upon the subjects of which it treats. Platina is largely used in dentistry, and its cost is so great that nothing which throws light upon the utilization of its residues can be considered superfluous.

